# Supplementary material for: High mRNA Expression Levels of Heat Shock Protein Family B Member 2 (HSPB2) Are Associated with Breast Cancer Patients’ Relapse and Poor Survival
Source: Int J Mol Sci. 2022 Aug 28;23(17):9758. doi: 10.3390/ijms23179758 (PMC9456243; doi:10.3390/ijms23179758)
Supplement: Supplementary file 1 [file ijms-23-09758-s001.zip › ijms-1867646-supplementary.pdf]

Supplementary Figures

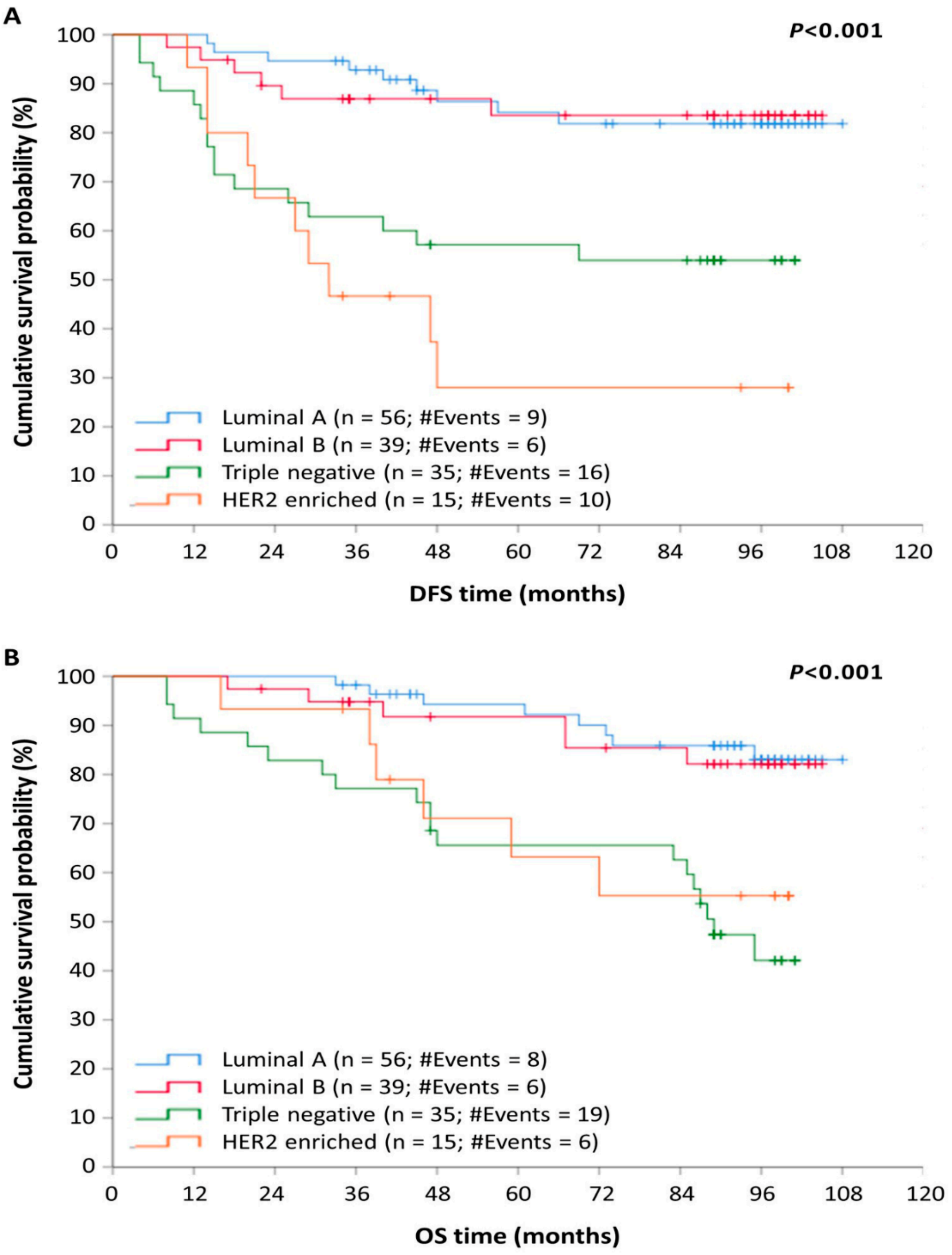

Figure S1: Stratified Kaplan-Meier survival curves, according to the molecular subtype, for disease-free survival (DFS) and overall survival (OS) of BrCa patients. Patients with triple negative or HER2 positive tumors had significantly lower DFS (**A**) and OS (**B**) rates intervals as compared with patients with luminal A or luminal B tumors. The *p* values were calculated using the log-rank test.

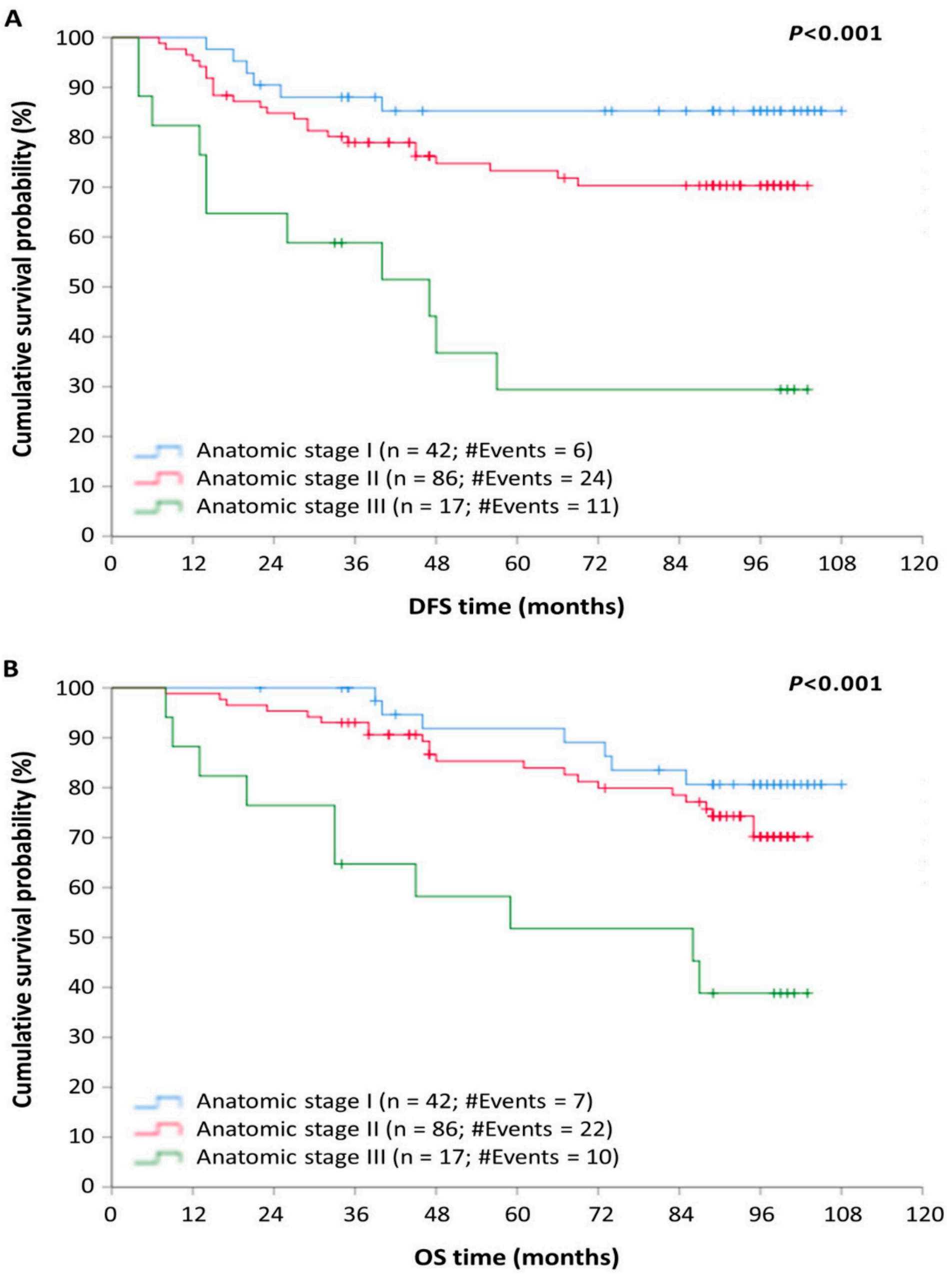

Figure S2: Stratified Kaplan-Meier survival curves, according to the anatomic stage, for disease-free survival (DFS) and overall survival (OS) of BrCa patients. Patients with tumors of anatomic stage III had remarkably shorter DFS (**A**) and OS (**B**) compared to those with tumors of anatomic stage I or II. The p values were calculated using the log-rank test.

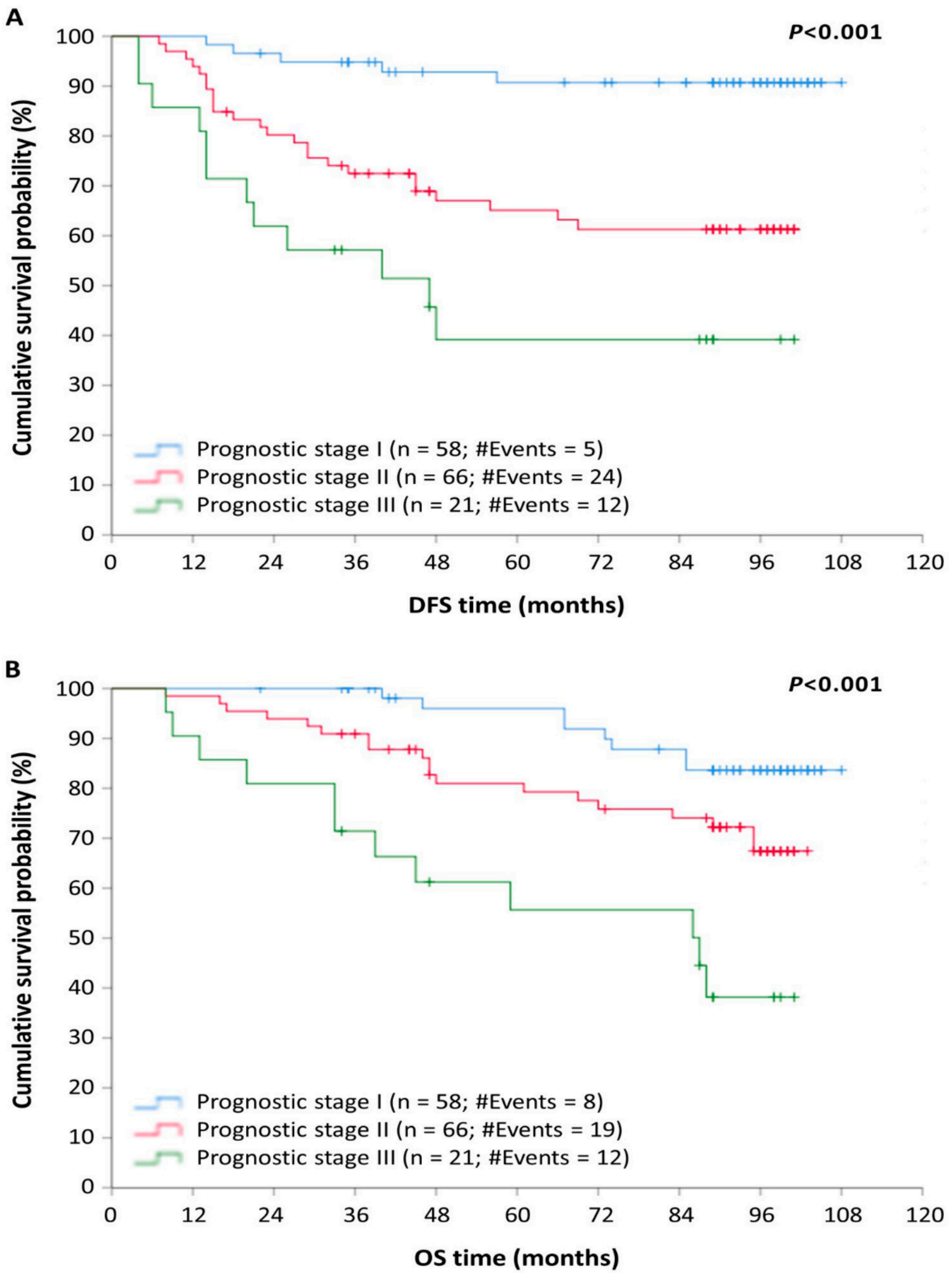

Figure S3: Stratified Kaplan-Meier survival curves, according to the prognostic stage, for disease-free survival (DFS) and overall survival (OS) of BrCa patients. Patients with prognostic stage III tumors were more likely to have poor outcomes, such as relapse (**A**) and death (**B**), as compared to patients with stage I or II tumors. The *p* values were calculated using the log-rank test.
